# Dendritic Cells and HIV-1 Trans-Infection

**DOI:** 10.3390/v2081704

**Published:** 2010-08-17

**Authors:** David McDonald

**Affiliations:** Department of Molecular Biology and Microbiology, Case Western Reserve University School of Medicine, Cleveland, OH 44106, USA; E-Mail: djm41@case.edu

**Keywords:** myeloid dendritic cell, C-type lectin receptor, antigen presentation, *trans*-infection, infectious synapse

## Abstract

Dendritic cells initiate and sustain immune responses by migrating to sites of pathogenic insult, transporting antigens to lymphoid tissues and signaling immune specific activation of T cells through the formation of the immunological synapse. Dendritic cells can also transfer intact, infectious HIV-1 to CD4 T cells through an analogous structure, the infectious synapse. This replication independent mode of HIV-1 transmission, known as *trans*-infection, greatly increases T cell infection *in vitro* and is thought to contribute to viral dissemination *in vivo*. This review outlines the recent data defining the mechanisms of *trans*-infection and provides a context for the potential contribution of *trans*-infection in HIV-1 disease.

## 1. Dendritic cells and immune control

Dendritic cells (DCs) comprise a diverse family of cell types whose primary function is to coordinate innate and adaptive immune responses. Classical DCs, commonly referred to as myeloid DCs (mDC), are essential antigen presenting cells that patrol submucosal surfaces in search of pathogenic organisms and their antigens, which they bind and internalize using a variety of surface receptors. The DCs carry the intact pathogens and antigens to draining lymphoid tissues and degrade them into antigenic peptides that are loaded onto HLA Class II molecules for presentation to CD4^+^ T cells [1]. In this capacity, mDCs act as a conduit between the periphery and lymphoid tissues to initiate immune control of mucosal infections. Langerhans cells (LC), also of the myeloid lineage, similarly act as sentinels of epidermal and mucosal epithelia, however they are largely confined to local sites of infection within the epithelial layer where they sustain innate and adaptive immune responses. The third major subtype of circulating DCs, plasmacytoid DCs (pDCs), are thought to derive from the lymphoid lineage and are particularly effective at initiating early responses to viral infections. pDCs secrete large amounts of type-I interferons and pro-inflammatory cytokines, signaling the recruitment of DCs and other immune effectors to the site of pathogen invasion. While all three DC subsets play important roles in immune control and pathogenesis in HIV-1 infection, this review will focus on the myeloid subset of DCs because of their peculiar ability to both initiate immunity to HIV-1 and to exacerbate HIV-1 infection by passing the intact virus to the very T cells responding to the insult. The origins and functions of dendritic cells is reviewed in great detail elsewhere, most notably by Geissmann [2] and Steinman [3].

When mDCs encounter and engulf pathogens at the site of infection, they undergo a series of maturation events and emigrate to local lymph nodes. Maturation is accompanied by activation of antigen degradation and presentation, up-regulation of T cell co-stimulatory molecules, and near complete shutdown of macropinocytosis and phagocytosis [4]. Once in the lymph node, the DCs present antigen/MHC Class-II complexes to CD4^+^ T cells to initiate adaptive immune responses to the pathogens they have carried from the periphery. Maturation freezes the expression of stabilized peptide/MHC-II complexes on the cell surface, prolonging presentation of peptides from antigens processed at the time of pathogen exposure, a model that has been termed the freeze-frame hypothesis. Because endocytosis is shut down, newly acquired antigens are not trafficked into the lysosomal degradative pathway for presentation, resulting in preferential presentation of antigens acquired at the site of peripheral tissue challenge [5–7].

When a CD4^+^ T cell encounters a DC in the lymph node, adhesion molecules such as LFA-1 and ICAM-1 cause the cell to arrest its movement and probe the DC surface. If the DC presents the correct antigen peptide/MHC-II complexes, the T cell will respond by concentrating T cell receptors, CD4 and numerous other adhesive and signaling molecules to form a structure known as the immunological synapse [8,9]. Antigen recognition by the T cell receptor stabilizes DC-T cell contacts that can last for several hours, providing sustained signals to the T cell to induce activation and proliferation [10]. In the absence of antigen recognition, incomplete immunological synapses are formed that generate weak signals thought to sustain T cell viability without activating the cells. In this capacity, DCs can help maintain naïve and memory T cells prior to antigenic challenge [11].

In addition to presentation of processed peptide antigens, DCs can transfer intact proteins and pathogens to other cells. In this capacity, DCs can transport antigens from sites of peripheral infection to lymphoid tissues, where they can pass them on to other antigen presenting cells and thereby enhance the breadth of immune responses [12]. DCs can also present intact antigens to B cells in lymphoid tissues to stimulate antigen specific proliferation and antibody secretion [13]. Both of these processes are mediated by endocytic uptake of antigens and accumulation into late endosomal structures known as multivesicular bodies or endosomes (MVB/MVE). MVBs are formed by inward budding of endosomes to the limiting membrane, resulting in the accumulation of “inside-out” endosomes approximately 50 nm in diameter called exosomes. After antigen uptake, MVBs can either traffic into the degradative lysosomal system to feed the antigen processing pathway or they can be routed back to the surface, where they deliver surface receptors back to the plasma membrane and excrete exosomes out of the cell (reviewed in [14,15]).

## 2. Role of dendritic cells in viral dissemination

Because of their crucial role in initiating innate and adaptive immune responses, DCs are often specifically targeted by viruses in what can be thought of as a form of offensive immune evasion. For example, mouse mammary tumor virus (MMTV), a milk transmitted retrovirus that ultimately infects mammary epithelial cells, establishes its initial reservoir in the gut of suckling mice by directly infecting DCs, which then carry the virus to lymphatic tissues. In the lymph node, the infected DCs activate T cells, which provide activating signals to DCs, B- and T cells. The activated lymphocytes and DCs provide a reservoir of infected and infection competent cells, which then carry the virus to mammary tissues. Importantly, ablation of dendritic cells prior to MMTV exposure blocks initial infection and DC ablation during ongoing MMTV infection attenuates the replication of the virus, indicating that DCs are likely involved both in initiating infectious events as well as ongoing viral amplification and dissemination throughout the course of infection [16,17]. Dendritic cells are targeted by numerous other viruses, either by direct infection or by immune modulation, often targeting the innate responses that are critical for DC mediated immune control. Herpesviruses, Papilloma and Foot- and-mouth disease viruses have all been shown to modulate DC function in order to enhance viral transmission and pathogenicity (reviewed in [18]). It is not surprising, then, that HIV-1 may have developed means to exploit dendritic cell mediated immune functions.

Because DCs reside in submucosal tissues, they are thought to be among the first cells that encounter HIV following sexual transmission. The three major types of tissue dendritic cells play distinct roles in HIV infection. Langerhans cells are susceptible to HIV-1 infection *in vitro* and can initiate robust infection of memory CD4 T cells [19,20], however LC appear to be largely resistant to HIV-1 infection and may instead act to bind and degrade the majority of incoming virions in the subepithelium [21]. Plasmacytoid DCs respond to localized HIV-1 infection in the submucosa and initiate inflammatory responses and recruitment of immune cells, and in particular CD4 T cells, which may fuel the early expansion of HIV-1 [22]. In addition, pDCs appear to be a major source of type 1 interferons that drive immune activation during pathogenic Simian Immunodeficiency Virus (SIV) infection [23].

Myeloid DCs are particularly resistant to infection by HIV-1 *in vitro* and productively infected mDCs have not been identified in infected individuals, likely due to their innate resistance to infection [24,25]. Interestingly, HIV-2 and SIV can overcome this innate resistance to infection by incorporation of viral vpx proteins into the virion core [26], and HIV-1 infection can be rescued by delivery of vpx into DCs by co-incubation with HIV-2/SIV viral-like particles, suggesting that vpx can counteract an antiviral restriction present in DCs [25]. The consequences of DC infection by SIV/HIV-2 has not yet been studied in detail, though it will no doubt provide insight into the contributions of *cis*- and *trans-*infections *in vivo*. The remainder of this review, however, will focus primarily on replication independent *trans*-infection by mDCs.

Myeloid dendritic cells were first implicated in HIV-1 pathogenesis nearly two decades ago when it was found that HIV-1 exposed DCs could greatly stimulate infection without first becoming infected and that purified DCs could nucleate clusters of CD4^+^ T cells and drive their infection *in vitro* [27,28]. These early studies suggested that HIV-1 could take advantage of the partnership between CD4 T cells and DCs, providing a microenvironment harboring infectious HIV-1 while at the same time increasing T cell susceptibility to infection. A key insight into the mechanism of HIV-1 transfer by DCs was the identification DC-SIGN (DC-specific ICAM-3 grabbing non-integrin, CD209), a C-type lectin receptor (CLR) that binds the HIV-1 envelope glycoprotein gp120 with high affinity [29]. DC-SIGN is known to function as an important adhesion receptor in DC trafficking and immune synapse formation and can also act to capture and internalize soluble antigens [30,31]. Surprisingly, when HIV-1 is bound by DC-SIGN, the virus remains infectious and can be efficiently transmitted to target cells without first replicating in the DC. This replication-independent transfer, referred to as *trans*-infection, requires gp120/CD4 mediated fusion into target cells, indicating that the virus must remain fully intact within the DCs. It has since become apparent that numerous factors can mediate or modulate *trans*-infection, including other C-type lectin receptors (CLRs) [32,33], heparin-sulphate binding syndecans [34] and even glycosphingolipid content of the virus [35].

## 3. HIV-1 *trans*-infection at the infectious synapse

Initial studies using *in vitro* cultured monocytes-derived dendritic cells (MDDCs) and cell lines expressing DC-SIGN demonstrated that DCs could sequester infectious HIV-1 for several days after uptake, leading to the hypothesis that DCs might initiate primary infections by carrying HIV-1 from mucosal sites of exposure to lymphoid tissues and transferring infection to CD4 T cells in the lymph node [29]. Moreover, the virus appeared to be sequestered within a protease resistant, endosomal compartment and trafficked back out of the cell prior to *trans*-infection [36], suggesting that HIV-1 could hide from the extracellular milieu within the DC and re-emerge on encounter with a target cell, a model referred to as the “Trojan horse” hypothesis. Direct visualization using GFP-tagged HIV-1 revealed that DCs *trans*-infect HIV-1 by binding and concentrating the intact virus at the cellular interface and at the same time induce recruitment of HIV-1 receptors CD4, CXCR4 and CCR5 on the T cell, forming a structure analogous to the immunological synapse and therefore called the infectious synapse [37]. A subsequent study demonstrated that infected T cells can transfer HIV-1 to other CD4 T cells by forming a similar structure, the virological synapse [38]. An important distinction between these two forms of contact mediated transmission is that virological synapse formation requires interaction of HIV-1 envelope glycoprotein on the infected cell surface with CD4 on the target, whereas DC *trans*-infection relies on adhesion molecules utilized during natural immune interactions [38–40]. DCs can be infected *in vitro*, albeit with very low efficiency, in which case HIV-1 gp120 dependent virological synapse formation results in *cis*-infection of T cells [41,42]. HIV-1 transmission in *cis*- and *trans*- appear to utilize similar but distinct pathways in DCs [43,44], an important consideration when evaluating experimental results that do not distinguish the two possibilities.

**Figure 1.**
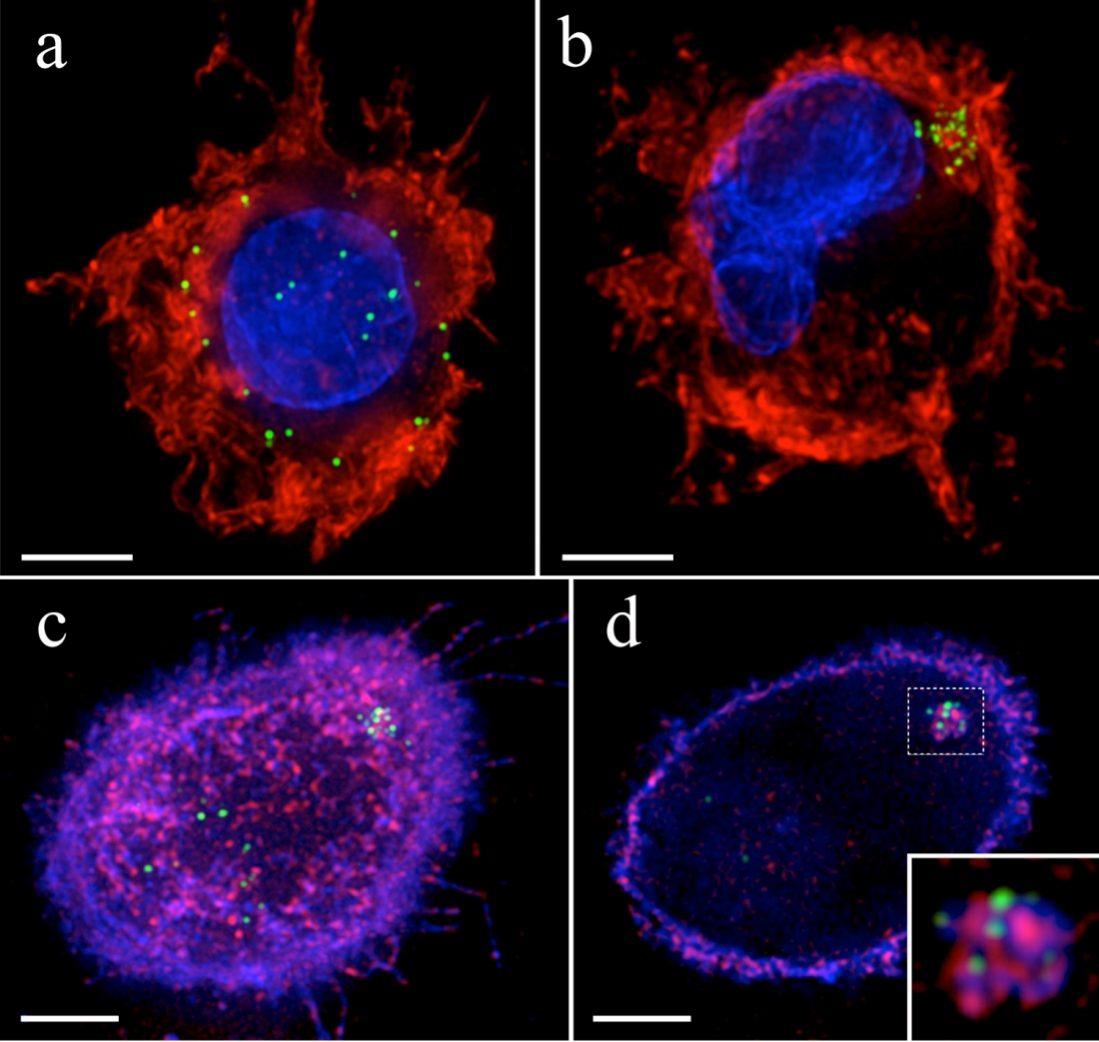
HIV-1 is sequestered into a distinct subcellular compartment in mature dendritic cells. **A-B**. Distribution of HIV-1 (green) in immature (**a**) and LPS matured (**b**) MDDCs. Images are whole cell volume rendered z-stacks, actin cytoskeleton (red) nuclear DNA (blue). **C-D**. Fluorescent 3D (**c**) and single z-plane (**d**) images of a mature DC harboring HIV-1 (green) stained for MHC-II (blue) and CD86 (red). Inset (**d**) shows magnified HIV-1 compartment. Bars, 5 μm.

Early studies demonstrated that prior maturation of the DCs with lipopolysaccharide (LPS) and other activators markedly enhances infectious synapse formation and concomitant *trans*-infection [37,45]. Indeed, immature DCs rapidly endocytose and degrade HIV-1 and may not harbor intact virus long enough to carry it to lymphatic tissues, as originally hypothesized [41,46]. Immature DCs efficiently present processed HIV-1 antigens to CD4^+^ T cells, indicating that HIV-1 does not subvert the Class II processing pathway in dendritic cells [47]. DC-SIGN and other scavenger receptors, therefore, appear to be primarily involved in virus uptake, endocytosis and degradation in immature DCs, and *trans*-infection may be a byproduct of incomplete internalization. LPS matured DCs, on the other hand, have greatly diminished capacity to degrade and present newly bound HIV-1 due to down-modulation of the endo-lysosomal system. Instead, mature DCs concentrate HIV-1 into a single compartment originally identified as an unconventional endocytic compartment rich in the tetraspanin CD81, a marker of multivesicular endosomes/MVBs, and lacking lysosomal/degradative markers such as CD63 and LAMP-1 [48]. A number of studies supported the notion that mature DCs sequester intact HIV-1 within an intracellular, MVB-like endosome that is protected from the extracellular mileau, and delivery to T cells at the infectious synapse is routed through the MVB/exocytosis pathway [49–52] (Figure 1). A conflicting study, however, suggested that HIV-1 contained within endosomes could not participate in *trans*-infection. Warner Greene’s group convincingly demonstrated that *trans*-infected HIV-1 was fully sensitive to surface-applied soluble CD4 (sCD4) even after the virus was sequestered within the DCs [53]. While it was already known that fusion inhibitors could block *trans*-infection when present during co-culture, this study showed that the virus was inactivated even when sCD4 was applied after HIV-1 binding and sequestration and washed away before co-culture. The inhibitors were added at 4 °C to prevent endocytosis, and therefore membrane impermeable inhibitors like sCD4 could not access endocytic compartments and should not be able to inhibit the sequestered HIV-1. Nevertheless, *trans*-infection was completely blocked, leading the authors to conclude that only surface-bound virions participated in *trans*-infection. Surface inhibition was demonstrated over a large range of input virus in *trans*-infection mediated by either immature or mature DCs. Intriguingly, a small amount of surface sCD4-resistant *trans*-infection occurred when antigen recognition was mimicked using Staphylococcus enterotoxin, however even under those conditions greater than 90% of virus transfer was inhibited. The authors concluded, therefore, that *trans*-infected HIV-1 was primarily derived from surface-bound virions, and that the sequestered, internalized HIV-1 was “dead-end” virus bound for degradation [53].

This was a surprising conclusion that conflicted with the fluorescent imaging data, and so we undertook a careful re-examination of *trans*-infection by mature DCs in light of these results [54]. Using time-lapse microscopy, we showed that the sequestered HIV-1 was rapidly recruited to sites of T cell contact, and individual particles trafficked out of the HIV-1 containing compartment and entered the T cell at the infectious synapse. Importantly, after transmission the virus rapidly fused into the T cell cytoplasm, directly demonstrating that HIV-1 delivery from intracellular stores resulted in productive entry of the viral particles at the infectious synapse. Remarkably, we found that although the sequestered HIV-1 appeared to be contained within intracellular compartments in the DCs, the structure remained contiguous with the cell surface and distinct from endocytic vesicles. Using several different membrane impermeable protein and lectin probes, we demonstrated that the HIV-1 residing within the DCs remained accessible to surface-applied ligands and that the compartment appeared to be comprised of invaginated plasma membrane folds. Therefore when inhibitors like sCD4 were added to the DCs it accessed the sequestered, “intracellular” HIV-1 and inactivated it. Importantly, if the DCs were fixed prior to application of the probes, the sequestered HIV-1 became inaccessible unless cellular membranes were first removed with detergent, suggesting that formaldehyde fixation “closed” the access to the surface-applied probes and that the connection to the surface was restricted in size. This is likely the reason we and others had concluded that the sequestered virus was contained within intracellular, endosomal compartments. Our results showed that the HIV-1 was in fact transferred from an internalized, endosome-like structure, however the structure remained connected to the cell surface, rendering the virus susceptible to surface-applied inhibitors [54]. Langerhans dendritic cells (LCs) appear to *trans*-infect HIV-1 through similar surface-exposed structures [53,55], however there is some conflicting evidence suggesting that LCs are protected from infection by expression of the CLR Langerin [21].

**Figure 2.**
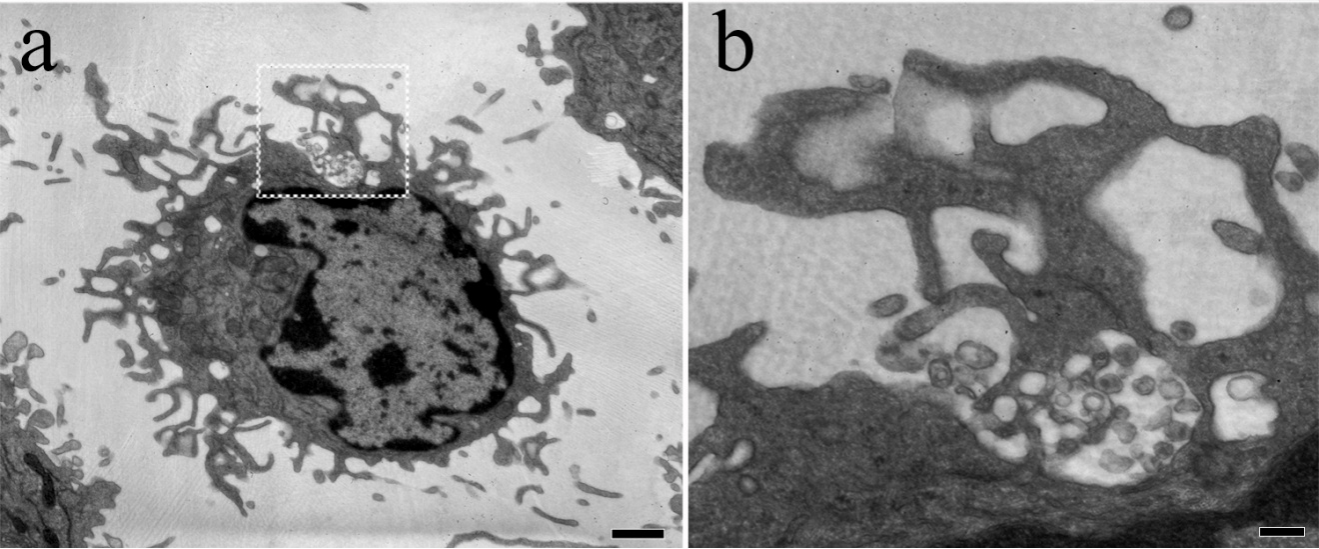
HIV-1 is trapped within surface-accessible membrane invaginations. **a**. Thin section transmission electron micrograph of a mature DC with HIV-1 particles trapped within apparent membrane folds. Cells were pulsed with HIV-1^LAI^, fixed, prepared and sectioned for TEM. **b**. Magnified view of boxed area in a. Bars, 1 μm (a), .2 μm (b).

Similar structures have been described in productively infected macrophages, which concentrate HIV-1 in CD81-positive compartments originally identified as late endosome/MVBs [56]. EM analysis revealed that the HIV-1-containing compartments in macrophages consist of a network of invaginated structures contiguous with the plasma membrane and accessible by surface applied, membrane impermeant probes [57,58]. Three dimensional reconstruction of serial EM sections showed that the virus was contained within surface-derived membrane folds [59] that appear surprisingly similar to structures that can be observed in mature DCs (Figure 2). Interestingly, sequestered HIV-1 is rapidly translocated to the virological synapse formed between infected macrophages and uninfected T cells [60], further suggesting commonality between macrophage and DC mediated HIV-1 transmission.

**Figure 3.**
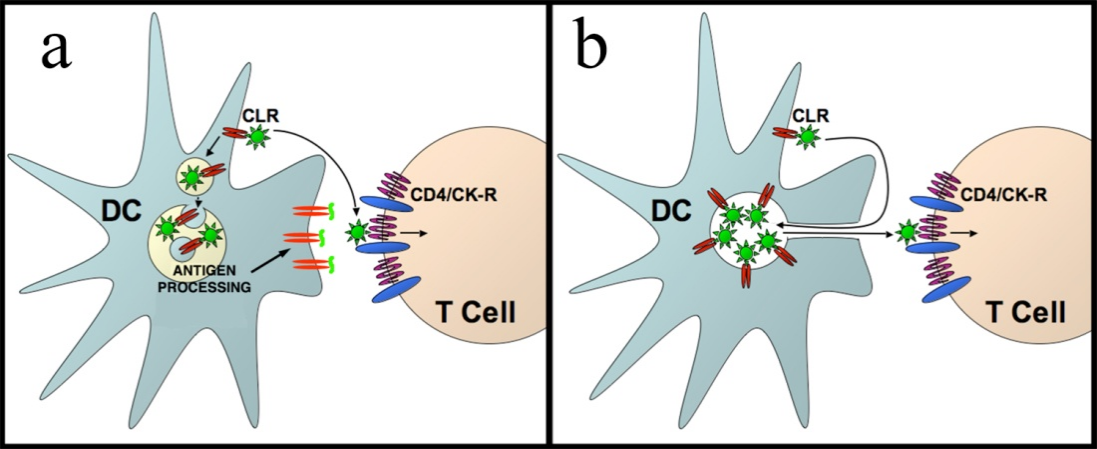
Models of immature and mature DC mediated *trans*-infection. **a**. The majority of HIV-1 bound by immature DCs is endocytosed, processed and presented as MHC-II/peptide complexes. HIV-1 remaining on the DC surface can be transmitted at the infectious synapse. **b**. Mature DCs concentrate viral particles within membrane folds. HIV-1 can emerge from the pocket structure after engaging CD4 on interacting T cells. CLR, C-type lectin receptor; CK-R, Chemokine receptor. Adapted from [54].

Taken together, these data suggest that immature dendritic cells efficiently endocytose, degrade and present HIV-1 antigens, and that *trans*-infection by immature DCs is a consequence of residual virus that remains on the cell surface. Mature DCs, on the other hand, have diminished capacity to endocytose the virus and instead form pocket-like compartment in DCs in order to remove infectious virions from the cell surface and to maintain them in an intact form during transport and delivery to other antigen presenting cells in lymphoid tissues. As a consequence, infectious HIV-1 can be delivered to CD4 T cells during immune presentation, resulting in inadvertent dissemination by the DCs (Figure 3). Intriguingly, our high-resolution imaging of DC-T cell conjugates suggests that the T cells may participate in *trans*-infection by extending membrane protrusions into the virus-containing pocket. Moreover, while both CD8 and CD4 T cells can extend these protrusions, virus particles transferred only into the CD4 cells, suggesting that HIV-1 can engage CD4 on the T cell protrusions to emerge from the pocket, consistent with the observation that contact with CD4 T cells facilitates the release of HIV from DCs at the infectious synapse whereas CD8 T cells does not [61]. These results could explain why HIV-1 egress from the pocket structure is relatively inefficient, however once it leaves the pocket the virus rapidly fuses into the T cell. The interacting T cells may play an important role in fishing the virus out of the pocket, and CD4 engagement of HIV-1 gp120 on the virion could favor trafficking along the T cell protrusions. HIV-1 uses the actin/myosin machinery to move on similar structures, referred to as cytonemes or nanotubes, during transmission from infected cells, [62,63], further suggesting there may be a common link between the virological and infectious synapses.

## 4. HIV-1 dissemination in lymphoid tissues

In recent years there has been a paradigm shift in our understanding of HIV pathogenesis, stemming from the recognition that the earliest targets of infection are mucosal memory CD4^+^ T cells. Simian models of HIV infection demonstrated that the virus establishes its initial reservoirs of infection in resting T cells of the gut associated lymphoid tissue (GALT), and subsequent immune activation due to damage to the gut establishes rampant infection and depletion of CD4^+^ T cells [64–66]. Similar massive T cell loss can be observed in biopsies of gut tissue from HIV-1-infected individuals [67]. In the SIV/macaque model, the earliest detectable targets of infection during vaginal transmission appear to be resting CD4 T cells in the lamina propria underlying the genital mucosa [64]. Plasmacytoid DCs (pDCs) appear to be the first cells that respond to infection in the lamina propria, and abrogating their recruitment can be protective to vaginal challenge [22]. Moreover, innate responses mediated primarily by pDCs distinguishes pathogenic and nonpathogenic SIV infections [23]. A potent immune response to primary infection therefore does not result in clearance of HIV-1 infection, but rather provides an ideal environment for establishing chronic infection, which in turn drives mucosal dendritic cells into lymphoid tissues and provides additional stimulus for viral amplification [68]. Activated DCs can amplify HIV-1 infection within lymphoid tissues by continually picking up and *trans*-infecting HIV-1 during the ongoing immune battle, and preferential infection of HIV-1 specific CD4 T cells may be a consequence of stabilized DC-T cell immune synapse formation leading to increased opportunity to pass on HIV-1 infection, resulting in their accelerated depletion and consequent failure to control infection [69,70].

## 5. Conclusions

DCs play a critical role in the establishment and persistence of viral infection in HIV disease. Because of their pivotal role in initiating immune responses, DC based immune therapies have been widely studied as potential therapeutic and vaccine vectors. Before DC therapies can be applied, however, it is essential to fully appreciate the role they play in HIV-1 dissemination. Clearly, *trans-*infection is an undesirable byproduct of viral clearance by DCs.

Chronic immune activation is a hallmark of HIV-1 infection, ultimately resulting in immune collapse and failure to control opportunistic infections. During the course of disease, the human immune system engages in a pitched battle with the virus, however the potent CD4^+^ T cell responses that are elicited are the very thing that provides the fuel for HIV-1 infection. The resultant downward spiral of CD4^+^ T cells ultimately leads to collapse of T cell populations and complete immune system failure. Strategies to boost immune control of HIV-1, therefore, must be directed at intervention of viral spread in the responding T cells, giving them a fighting chance to control viremia. In the course of a single day, one DC can interact with dozens of T cells in a lymph node, providing ample opportunity to transmit HIV-1 to either antigen-activated or quiescent T cells. Therefore, DCs can act as a replication-independent viral reservoir that is protected from attack both by current anti-viral drug regimens and by the host defense systems. There appears to be a dynamic balance between antigen processing and *trans*-infection in DCs, and putting the brakes on *trans*-infection may be one way to shift the balance to favor immune control of HIV-1 disease.
